# Assessment of the Effects of Double-Stranded RNAs Corresponding to Multiple Vitellogenesis-Inhibiting Hormone Subtype I Peptides in Subadult Female Whiteleg Shrimp, *Litopenaeus vannamei*

**DOI:** 10.3389/fendo.2021.594001

**Published:** 2021-03-02

**Authors:** Bong Jung Kang, Zakea Sultana, Marcy N. Wilder

**Affiliations:** Fisheries Division, Japan International Research Center for Agricultural Sciences, Tsukuba, Japan

**Keywords:** double-stranded RNA, *Litopenaeus vannamei*, RNA interference, vitellogenesis-inhibiting hormone, vitellogenin

## Abstract

Vitellogenesis-inhibiting hormone (VIH) negatively regulates reproduction in shrimp and other decapod crustaceans. In order to assess the effects of transcriptional silencing by multiple VIH subtype I sinus gland peptides (SGPs) on ovarian maturation in female whiteleg shrimp, *Litopenaeus vannamei*, we synthesized five dsRNAs targeting *Liv-SGP-A*, −*B*, −*C*, −*F*, and −*G* and injected them into subadults. The following treatments were employed: sgpG-dsRNA (targeting *Liv-SGP-G*), sgpC-dsRNA (targeting *Liv-SGP-C*), and mixed-dsRNA (targeting *Liv-SGP-A*, *−B*, and *−F*). The expression of *Liv-SGP-G* in eyestalks was significantly decreased at 10, 20, and 30 days after the injection of sgpG-dsRNA In addition, it was significantly decreased at 10 and 30 days after the injection of mixed-dsRNA. The expression of vitellogenin (Vg) gene expression in the ovaries, and concentrations of Vg protein in the hemolymph, were not changed by the administration of any dsRNA treatment (the ovaries remained immature in all treated individuals and contained mostly oogonia and previtellogenic oocytes). Although the administration of dsRNAs corresponding to multiple VIHs did not promote ovarian maturation, this is the first report of the co-transcriptional repression of *Liv-SGP-G* by the injection of dsRNA for homologous genes (*Liv-SGP-A*, −*B*, and *−F*). These results indicate that subadults can respond to the techniques of transcriptional silencing.

## Introduction

The hormonal regulation of ovarian maturation in decapod Crustacea is more well-understood in relation to inhibitory factors than to stimulatory factors. Eyestalk ablation is frequently employed as an artificial means of promoting ovarian maturation in economically important shrimp species, and is frequently used in commercial hatcheries to induce spawning (1–3). For this reason, it had long been assumed that the eyestalk harbors a maturation-inhibiting factor, now referred to as vitellogenesis-inhibiting hormone (VIH; also gonad-inhibiting hormone, GIH). There now exist many reports regarding its physiological functioning, including information on its identification and characterization (4–7). VIH is expressed and synthesized at the X-organs and stored in the sinus glands in the eyestalks; these tissues are collectively referred to as the X-organ/sinus gland complex. VIH thus synthesized is secreted into the hemolymph and negatively regulates ovarian maturation. In *Litopenaeus vannamei* and other penaeid shrimp species, VIH comprises a set of peptide hormones that belong to the crustacean hyperglycemic hormone (CHH) family. CHH-family neuropeptides also include CHH, VIH, molt-inhibiting hormone (MIH), and mandibular organ-inhibiting hormone (MOIH). Most CHH-family peptides are synthesized at and secreted predominantly from the X-organ/sinus gland complex, but some are synthesized at other tissues (8–11). Mature CHH-family peptides all have six conserved cysteine positions, and are divided into two subtypes depending on the absence (subtype I) or presence (subtype II) of a glycine residue at position 12. Generally, CHH is of subtype I, and VIH, MIH, and MOIH are of subtype II; however, subtype I VIH has also been reported in the penaeid shrimps *Penaeus japonicus* and *L. vannamei* (4–7).

Penaeid shrimp species are commercially important in shrimp farming worldwide; in particular, the whiteleg shrimp, *L. vannamei*, accounts for over 70% of farmed shrimp (12). Eyestalk ablation is routinely performed in adult females in commercial hatcheries in order to induce ovarian maturation, but the procedure often promotes adverse effects on the animals, lowers reproductive efficiency, and poses concerns for animal welfare (13). Thus, alternative methods based on the physiological functioning of VIH should be sought. The use of RNA interference (RNAi) has been considered as an alternative to eyestalk ablation where the administration of double-stranded RNA (dsRNA) silences the transcription of the vitellogenesis-inhibiting hormone gene *vih*; such work has been carried out in *Penaeus monodon* and *L. vannamei* (11, 14–16).

Both VIH subtype I and II are present in *L. vannamei*. As delineated in Tsutsui et al. (7), subtype I includes several peptides identified from the sinus glands, referred to as sinus gland peptides (SGPs)-A to −G, six of which (except SGP-D) possess VIH activity. We subsequently conducted the molecular characterization of five of these (*Liv-SGP-A, −B, −C, −F, −G*) and investigated their expression levels in relation to molting and eyestalk ablation (17, 18). In addition, we achieved the knockdown of the most predominant VIH subtype I (SGP-G) in adult *L. vannamei via* VIH-dsRNA injection into adult females, but this did not lead to an increase in vitellogenin (Vg) gene expression in the ovary and levels of Vg in the hemolymph even 20 days after injection (16). On the other hand, in Chen et al. (10) a gene for a VIH subtype II in *L. vannamei* was cloned from the eyestalks and brain, and recombinant peptide corresponding to VIH subtype II inhibited *vg* mRNA expression (10). Feijo et al. (15) reported the knockdown of GIH (VIH subtype II) transcription *via* GIH-dsRNA injection, with *vg* mRNA expression in the ovary being increased 37 days after injection (15).

Previously, we analyzed gene expression levels for VIH subtype I peptides in the eyestalks following unilateral eyestalk ablation in adult and subadult *L. vannamei*, and found a significant decrease only in subadults; specifically, the expression of *Liv-SGP-A*, −*C*, and −*G* decreased 10 or 20 days after eyestalk ablation (18). The question still remained as to why a significant reduction of *vih* expression following eyestalk ablation was shown only in subadults and for only three among five of the VIH species (e.g., Liv-SGP-A, -B, and -G as listed above).

In *L. vannamei*, subadult females can also be induced to undergo ovarian maturation using eyestalk ablation; therefore, such females are often used as experimental material in order to study the mechanisms of ovarian maturation (10, 19, 20). In this study, we employed subadult females in order to study in more detail the action of VIH on shrimp ovarian maturation at a very basic level in the absence of complications that may be observed in the adult system. We therefore suggest that if the inhibiting action of VIH on ovarian maturation is more powerful in subadults than in adults, the knockdown of VIH expression may be more effective in subadults. Therefore, here we have selected subadult female *L. vannamei* in order to assess the effects of multiple dsRNAs on the transcriptional silencing of *vih* genes, and to shed further light on the dynamics of *vih* transcription in early-stage shrimp, with the aim of better understanding the phenomenon of ovarian maturation. Therefore, in regard to the above, we aimed to examine the co-transcriptional repression of *Liv-SGP-G* based on the administration of dsRNA corresponding to multiple VIH subtype I peptides having differing degrees of similarity; this is first report of this kind for a decapod crustacean species.

## Materials and Methods

### Animals

Subadult female *L. vannamei* were purchased from IMT Engineering Inc. (Niigata Prefecture, Japan) and were gradually acclimated to recirculated natural seawater (31–33‰ salinity) after which they were kept in 600-L tanks at 28°C under a 13-h light/11-h dark photoperiod for 2 weeks before use in experimentation. All shrimp were fed with a commercial diet (Goldprawn; Higashimaru Co., Kagoshima, Japan) at a rate of 2–3% body weight per day until use. The treatment of all animals complied with institute regulations and Japanese policy on animal use (21).

### Preparation of Double-Stranded RNA

dsRNA targeting *vih* genes in *L. vannamei* (*Liv-SGP-A*, *−B*, *−C*, *−F*, and −*G*) and the gene for green fluorescent protein (GFP) as a negative control were prepared as previously elaborated (16). T7 promoter-linked linear DNA for each gene was amplified by PCR using the following T7 promoter-linked gene-specific primers: T7-sgpC-L (5′-TAATACGACTCACTATAGGGAGACT- CGCTCTTCGACCCTTCC-3′) and T7 promoter (5′-TAATACGACTCACTATAGGG-3′) for *Liv-SGP-A*; T7-sgpB-L2 (5′-TAATACGACTCACTATAGGGAGACGCAGCATATCCTTCGACTCGT-3′) and T7 promoter for *Liv-SGP-B*; T7-sgpC-L and T7-sgpC-R (5′-TAATACGACTCACTATAGGGAGACTATTTCCCGACCATCTGG-3′) for *Liv-SGP-C*; T7-sgpF-L (5′-TAATACGACTCACTATAGGGAGAAAGCGCTCCCTCTTCGACC-3′) and T7-sgpF-R (5′-TAATACGACTCACTATAGGGAGACTTTATTTGCCGACGGTCTGCAGG-3′) for *Liv-SGP-F*; and T7-VIH-L (5′-TAATACGACTCACTATAGGGAGAAAGCGAGCAAACTTCGAC-3′) and T7-VIH-R (5′-TAATACGACTCACTATAGGGAGACTACTTGCCCACCGTCTG-3′) for *Liv-SGP-G*. DNA fragments that encoded each mature peptide were purified and used to synthesize each dsRNA (sgpA-dsRNA for *Liv-SGP-A*, sgpB-dsRNA for *Liv-SGP-B*, sgpC-dsRNA for *Liv-SGP-C*, sgpF-dsRNA for *Liv-SGP-F*, sgpG-dsRNA for *Liv-SGP-G*, and GFP-dsRNA for *GFP*), following the protocol described in Kang et al. (16) using the MEGAscript RNAi kit (Ambion, Thermo Fisher Scientific, Tokyo, Japan) according to the manufacturer’s instructions.

### Injection of Double-Stranded RNA and Tissue Collection

sgpG-dsRNA, sgpC-dsRNA, and GFP-dsRNA were diluted with elution buffer (TE buffer: 10 mM Tris-HCl [pH 7], 1 mM EDTA) to 0.75 µg µl^−1^. Mixed dsRNA was prepared using three separate dsRNA mixtures having equivalent concentrations of sgpA-dsRNA, sgpB-dsRNA, and sgpF-dsRNA (each dsRNA concentration was 0.25 µg µl^−1^). This was diluted with TE buffer to yield 0.75 µg µl^−1^ total dsRNA. We next randomly selected 80 female shrimp having a body weight of above 15 g and injected 18 of them each with one of the above four dsRNA preparations at 3 µg g^−1^ body weight as in our previous report (16). In brief, all female shrimps were injected once only in the first abdominal segment intraperitoneally using a BD Ultra-Fine Insulin Syringe (0.3 ml, 29 G × 12.7 mm; BD Biosciences, Tokyo, Japan). Four dsRNA treatment groups were designed as follows: a single injection of either GFP-dsRNA (GFP-dsRNA group), sgpG-dsRNA (sgpG-dsRNA group), sgpC-dsRNA (sgpC-dsRNA group), or mixed-dsRNA (mixed-dsRNA group). All injected shrimps were maintained under the conditions delineated in the *Animals* section. We sampled six individuals from each treatment at 10, 20, and 30 days after injection and collected samples of hemolymph, eyestalk, ovary, and hepatopancreas as described previously (16). Body weight in each group are shown in **Table 1**. As the initial control, six non-treatment shrimps were also collected (body weights, mean ± SEM: 23.0 ± 1.23 g, n = 6). The gonadosomatic index (GSI) was calculated as gonad weight (g)/body weight (g) × 100 and expressed in terms of percentage. Since the number of shrimps that can be reared simultaneously under our recirculating rearing system poses certain limitations, in order to compare each treatment in an equivalent manner, six individuals for each treatment was the maximum number that could be employed under our rearing system. Statistically, n = 5 is generally considered acceptable, and in this way, we believe that our methodology is valid.

**Table 1:** Average body weight of treated shrimps (g, mean ± SEM, *n* = 6) at 0, 10, 20, and 30 days after treatment with each dsRNA preparation.

dsRNA prep.	10 days	20 days	30 days
GFP-dsRNA	21.2 ± 0.88	26.1 ± 1.90	28.8 ± 0.91
sgpG-dsRNA	23.5 ± 0.72	26.8 ± 0.96	27.4 ± 0.67
sgpC-dsRNA	23.6 ± 1.15	26.4 ± 1.20	27.7 ± 1.77
mixed-dsRNA	23.0 ± 0.89	25.4 ± 1.18	28.3 ± 1.08

### Quantitative Real-Time PCR for *vih* and *vg*

Total RNA was purified from eyestalks, ovaries, and hepatopancreas tissue using an RNeasy Mini kit with DNase I digestion according to the manufacturer’s protocol (Qiagen, Tokyo, Japan). In order to maximize the quality of RNA for subsequent qPCR analysis, the purified eyestalk total RNA was purified once more using an RNeasy MinElute cleanup kit (Qiagen) according to the manufacturer’s protocol. The relative expression of *Liv-SGP-G* was quantified by two-step qPCR as reported previously (16). In brief, the twice-purified eyestalk total RNA was used for reverse-transcription (RT) with a High Capacity RNA-to-cDNA kit (Applied Biosystems, Thermo Fisher Scientific, Tokyo, Japan) in accordance with the manufacturer’s protocol, and the synthesized cDNA was used as a template for qPCR. qPCR for *Liv-SGP-G* and *beta-actin* (*actb*, as an internal control) was performed with TaqMan Fast Universal PCR Master Mix (2×, No AmpErase UNG; Thermo Fisher Scientific) on a Model 7500 Fast Real-Time PCR System (Applied Biosystems, Foster City, CA, USA) as previously reported (16, 18), using the primer pair LvsgpG-Fw and LvsgpG-R3 and the TaqMan probe LvsgpG-Prb for *Liv-SGP-G*; and the primer pair Lvact_F01 and Lvact_R02 and the TaqMan probe Lvact_Prb for *actb*. The standard curves for *Liv-SGP-G* and *actb* were linear over five orders of magnitude of serially diluted cDNA libraries (eyestalk cDNA for *Liv-SGP-G*, testis cDNA for *actb*) (16). Expression was quantified in duplicate, and the mean of *Liv-SGP-G* was normalized to that of *actb*.

The relative expression of *Liv-SGP-A*, *-B*, and *-C* was also quantified by two-step qPCR as reported previously (18). In brief, the synthesized cDNA from twice-purified eyestalk total RNA was used as a template for qPCR. qPCR for *Liv-SGP-A*, *-B*, *-C* and *beta-actin* (*actb*, as an internal control) was performed using the same protocols of qPCR for *Liv-SGP-G*, but using the following gene-specific primers and TaqMan probes: SgpA-Fw, SgpA-Rv, and SgpA-Prb for *Liv-SGP-A*; SgpB-L2, SgpB-Rv, and SgpB-Prb for *Liv-SGP-B*; and LvsgpC-Fw, LvsgpC-Rv, and LvsgpC-Prb for *Liv-SGP-C*. The standard curves for *Liv-SGP-A*, *-B*, and *-C* were linear over seven orders of magnitude of serially diluted 4 ng of each plasmid (18). Expression was quantified in duplicate, and the mean of each gene expression data point was normalized to that of *actb*.

The expression of *vg* in the ovary and hepatopancreas was quantified by one-step real-time RT-PCR using a QuantiFast Probe RT-PCR + ROX Vial Kit (Qiagen) with purified total RNA as the template. The expression of *vg* and *actb* mRNAs was quantified on a Model 7500 Fast Real-Time PCR System as previously reported (16, 19), using the primer pair vg-qF01 and vg-qR01 and the TaqMan probe vg-Prb for *vg*; and the primer pair Lvact_F01 and Lvact_R02 and the TaqMan probe Lvact_Prb for *actb* (16). The standard curves for *vg* and *actb* were linear over five orders of magnitude of serially diluted total RNA from mature ovary. Expression was quantified in duplicate, and the mean of *vg* expression was normalized to that of *actb*.

### Measurement of Vitellogenin Concentrations in Hemolymph

Hemolymph Vg was measured by time-resolved fluoroimmunoassay (TR-FIA) as previously reported (19). In brief, each hemolymph sample was directly diluted 1:2,000 with 0.1 M carbonate buffer (CB, pH 9.6), and 100 μl of diluted sample was dispensed into the wells of 96-well plates (Delfia Yellow; Perkin Elmer, Waltham, MA, USA). To construct standard curves, purified vitellin from *L. vannamei* was serially diluted in negative buffer (male hemolymph diluted 1:2,000 in CB) to 50.1 to 0.10 ng per well for the TR-FIA Vg assay. For sample-coating, the 96-well plates were incubated at 4°C overnight with 100 μl of sample or standard, and then the wells were blocked with 1% BSA in assay buffer (Perkin Elmer). After blocking, vitellin antiserum from *Penaeus japonicus* (anti-PjVn) was diluted 1:10,000 in assay buffer for use as the primary antibody. Secondary antibody was Delfia Eu-N1-labeled anti-rabbit antibody (Perkin Elmer), diluted 1:2,000 in assay buffer. Plates were incubated following each step for 2 h at 24°C, and the wells were washed five times for 3 min at a time with 0.3 ml of washing buffer (50 mM Tris·HCl, pH 7.8, 150 mM NaCl, 0.05% Tween 20) in an ImmunoWash 1575 Microplate Washer (Bio-Rad) each time between steps. Finally, enhancement solution (Perkin Elmer) was added to the plate, and the fluorescence was measured at 615 nm on a time-resolved fluorometer (Wallac 1420 ARVOsx-d; Perkin Elmer).

### Histological Analysis

A small piece of ovary from each sampled individual was fixed in Davidson’s fixation solution (formalin, 220 ml L^−1^; EtOH, 330 ml L^−1^ 95%; acetic acid, 115 ml L^−1^) for 16–20 h and then held in 70% ethanol at room temperature before processing for histological analysis. Tissues were dehydrated through a graded series of ethanol and lemosol (Fujifilm Wako Pure Chemical Corporation, Tokyo, Japan), and then embedded in paraffin. Tissues were sectioned to 5 µm, hydrated through a graded ethanol series, stained with hematoxylin and eosin, and again dehydrated through a graded ethanol series and xylene. Oocyte developmental stage, determined according to previous reports, was observed under a light microscope (19).

### Statistics

Significant outliers among each group were identified by Grubbs’s test for outliers (α = 0.01) on the GraphPad website (https://www.graphpad.com/quickcalcs/Grubbs1.cfm) and omitted. All results are expressed as the mean ± SEM. Significant differences were assessed by one-way ANOVA using SigmaPlot v. 11 software (Hulinks Inc., Tokyo, Japan). The significance between *vg* expression levels and Vg concentrations in all groups was tested by multiple comparisons between the untreated (initial) group and treatment groups by Dunnett’s method. The significance of expression levels of *Liv-SGP-G* among individuals was tested by multiple comparisons between the initial group and treatment groups by Kruskal–Wallis ANOVA on ranks using Dunn’s method.

## Results

### Effects of the Injection of Multiple Double-Stranded RNAs on the Expression of *Liv-SGP-G*

**Figure 1:**
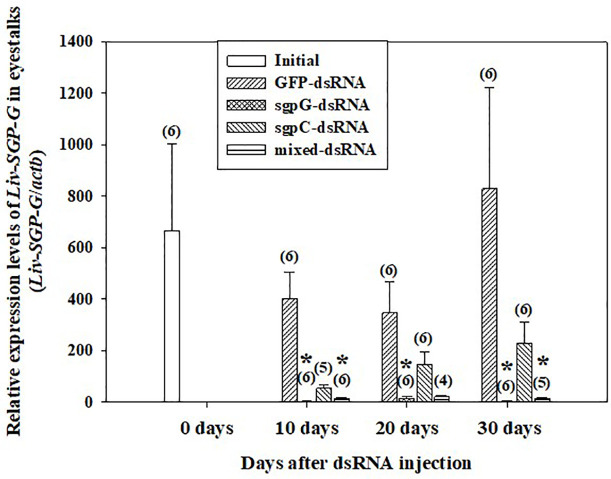
Levels of *Liv-SGP-G* expression in eyestalks following a single injection of GFP-dsRNA, sgpG-dsRNA, sgpC-dsRNA, mixed-dsRNA, or no injection (initial). Results are expressed as the mean ± SEM. Asterisks indicate significant difference (*P* < 0.05) from the initial group. Values in parentheses indicate numbers of shrimp analyzed in each group. *Beta-actin* is indicated as *actb*. The content of each treatment group is indicated in the legend box that appears above the bar graph.

**Figure 1** shows relative *Liv-SGP-G* expression levels in the eyestalk. In shrimps injected with GFP-dsRNA, *Liv-SGP-G* expression was high at 10 (*Liv-SGP-G* levels 400.7 ± 104.0) to 30 days (828.9 ± 393.6) after injection, with no significant difference from the initial group (664.0 ± 337.9). In the sgpG-dsRNA group, expression was significantly lower than that of the initial group at 10 days (3.011 ± 1.413) to 30 days (3.052 ± 0.943). In the sgpC-dsRNA group, expression was lower, but not significantly, than that in the initial group at 10 days (54.08 ± 13.14), and then increased gradually from 20 days (145.7 ± 51.31) to 30 days (229.0 ± 83.46). In the mixed-dsRNA group, expression was significantly lower than that in the initial group at 10 days (14.91 ± 2.379), lower (but not significantly) at 20 days (20.89 ± 5.447), and then significantly lower at 30 days (14.21 ± 2.830).

### Dynamics of *vg* Expression and Concentrations Following Double-Stranded RNA Injections

**Figure 2:**
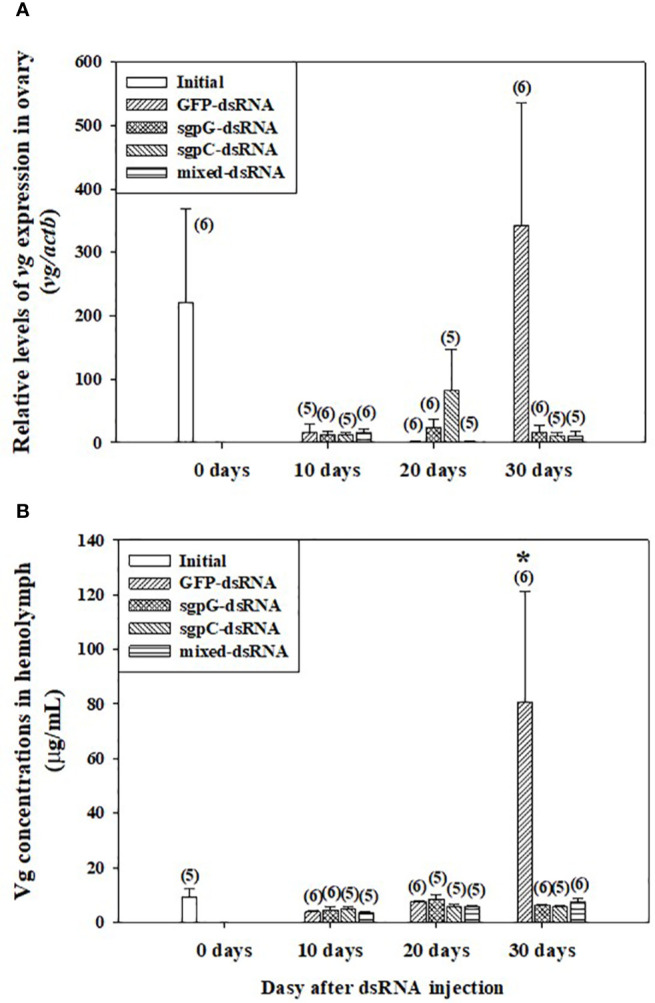
Levels of **(A)** *vg* mRNA in ovary and **(B)** Vg in hemolymph following a single injection of GFP-dsRNA, sgpG-dsRNA, sgpC-dsRNA, mixed-dsRNA, or no injection (initial). Results are expressed as the mean ± SEM. The asterisk indicates significant difference (*P* < 0.05) from the initial group. Values in parentheses indicate numbers of shrimp analyzed in each group. Abbreviations are as follows. Vg, vitellogenin; actb, beta-actin. The content of each treatment group is indicated in the legend box that appears above each bar graph.

**Figure 2A** shows relative *vg* mRNA expression in the ovary. In the initial group, expression was weak (four out of six shrimps) or easily detectable (two out of six shrimps) (*vg* mRNA levels 220.6 ± 147.8). In the GFP-dsRNA group, expression was weak at 10 days (15.16 ± 14.08) and 20 days (1.408 ± 0.578), and then strong at 30 days (343.1 ± 193.1), but with no significant difference compared to the initial group owing to high variation in measured values. In the other groups, *vg* mRNA expression was weak. Expression of *vg* mRNA in the hepatopancreas was weak or not detectable in all treatment groups, including the initial group (data not shown).

**Figure 2B** shows Vg levels in the hemolymph. In the initial group, levels were low (8.56 ± 1.82 µg ml^−1^). In the GFP-dsRNA group, levels were low at 10 days (3.76 ± 0.801 µg ml^−1^) and 20 days (7.51 ± 0.573 µg ml^−1^), with no significant difference observed from the initial group, but were significantly increased at 30 days (80.7 ± 40.6 µg ml^−1^). Levels in the other groups from 10 to 30 days were not significantly different from those of the initial group.

### Gonadosomatic Index and Oocyte Development

**Figure 3:**
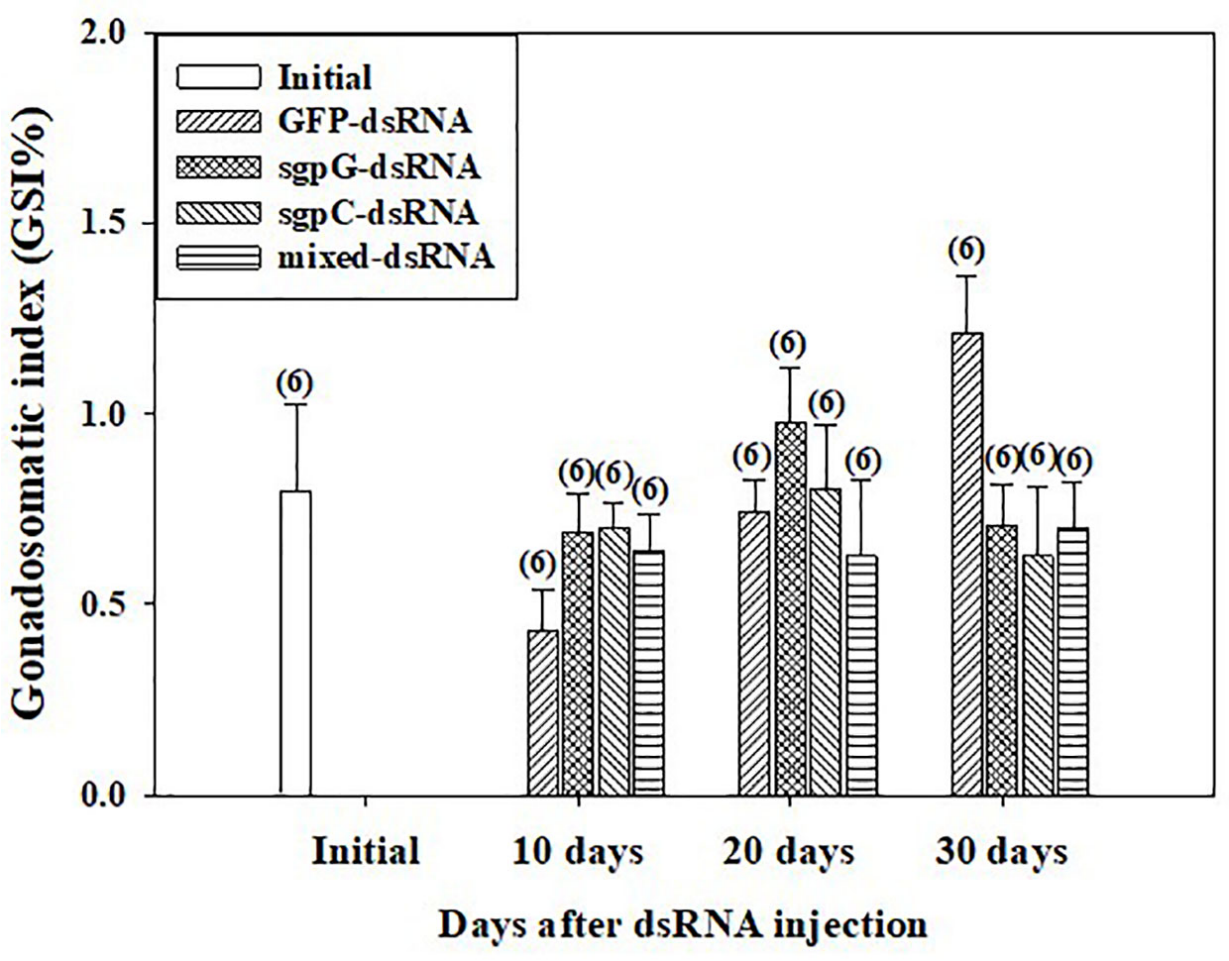
Changes in gonadosomatic index (GSI) following a single injection of GFP-dsRNA, sgpG-dsRNA, sgpC-dsRNA, mixed-dsRNA, or no injection (initial). Results are expressed as the mean ± SEM. Values in parentheses indicate numbers of shrimp analyzed in each group. The content of each treatment group is indicated in the legend box that appears above the bar graph.

GSI was low (<2%) from 10 to 30 days in all individuals (**Figure 3**), with no significant difference observed compared to the initial group (0.80 ± 0.23%). GSI tended to gradually increase from 10 days to 30 days in the GFP-dsRNA injected groups. In the sgpG-dsRNA injected group, GSI increased slightly from 10 days to 20 days and then decreased at 30 days.

**Figure 4:**
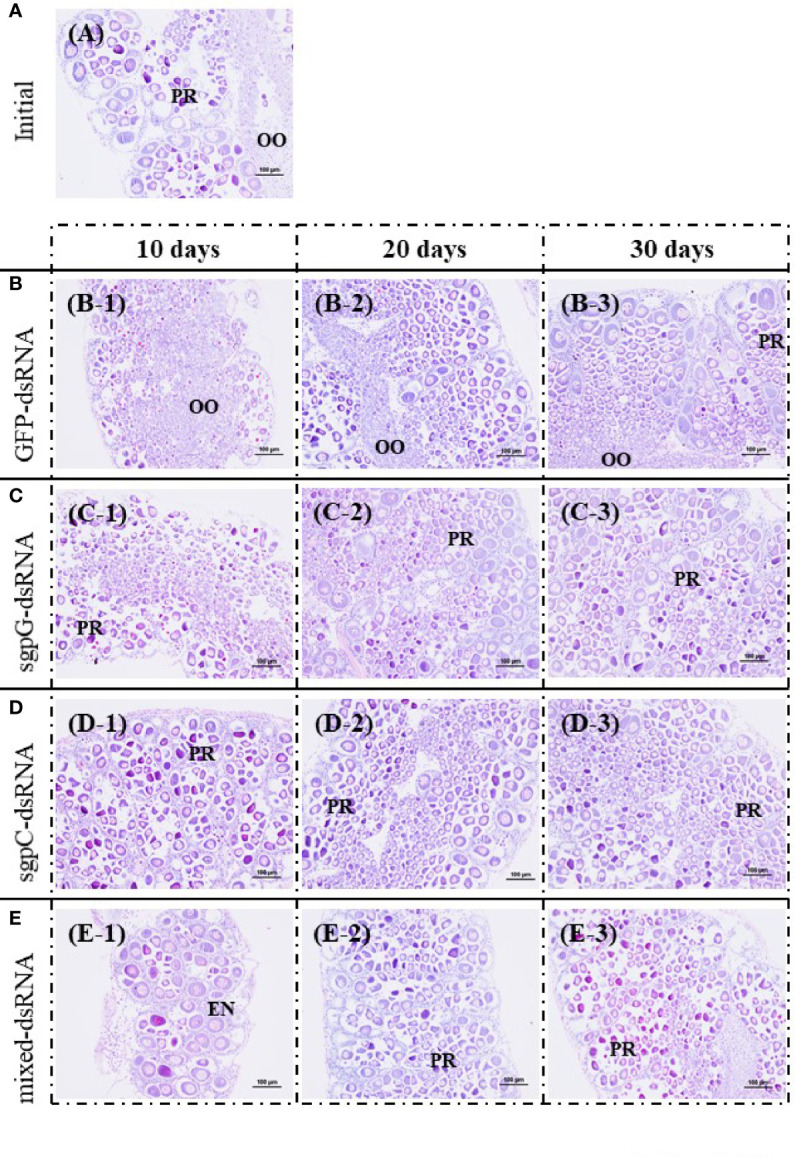
Histological images of oocyte development from representative individuals exhibiting a GSI close to the mean of each group, stained with hematoxylin and eosin. **(A)** Initial (no injection); A, gonadosomatic index (GSI) = 0.94%. **(B)** GFP-dsRNA; B-1, 10 days, GSI = 0.33%; B-2, 20 days, GSI = 0.70%; B-3, 30 days, GSI = 1.3%. **(C)** sgpG-dsRNA; C-1, 10 days, GSI = 0.69%; C-2, 20 days, GSI = 1.1%; C-3, 30 days, GSI = 0.73%. **(D)** sgpC-dsRNA; D-1, 10 days, GSI = 0.64%; D-2, 20 days, GSI = 0.82%; D-3, 30 days, GSI = 0.65%. **(E)** Mixed-dsRNA; E-1, 10 days, GSI = 0.64%; E-2, 20 days, GSI = 0.77%; E-3, 30 days, GSI = 0.62%. Oocyte developmental features: OO, oogonium; PR, previtellogenic oocytes; EN, endogenous vitellogenic oocytes. Bars = 100 µm.

**Figure 4** shows histological images of oocyte development from representative individuals exhibiting a GSI close to the mean of each group. In the initial group, oocytes in five of the six shrimps were in the previtellogenic stage, and contained mostly oogonia and previtellogenic oocytes (GSI = 0.94%, **Figure 4A**); only one shrimp exhibited an ovary in the primary vitellogenic stage (GSI = 1.9%). In the GFP-dsRNA group, ovaries were in the previtellogenic stage in all six shrimps at 10 days (GSI = 0.33%, **Figure 4B-1**) and 20 days (GSI = 0.70%; **Figure 4B-2**), and in five of the six shrimps at 30 days, two of which also had a few early-stage endogenous vitellogenic oocytes (GSI = 1.3%, **Figure 4B-3**); ovaries in the other shrimp were in the primary vitellogenic stage, containing endogenous vitellogenic oocytes (GSI = 1.9%).

In the sgpG-dsRNA group, ovaries were mostly in the previtellogenic stage in all six shrimps at 10 days (GSI = 0.69%, **Figure 4C-1**), 20 days (GSI = 1.1%, **Figure 4C-2**), and 30 days (GSI = 0.73%, **Figure 4C-3**). In the sgpC-dsRNA group, ovaries were mostly in the previtellogenic stage in all six shrimps at 10 days (GSI = 0.64%, **Figure 4D-1**), at 20 days (GSI = 0.82%, **Figure 4D-2**), one of which also possessed a few early-stage endogenous vitellogenic oocytes, and in all six shrimps at 30 days (GSI = 0.65%, **Figure 4D-3**). In the mixed-dsRNA group, ovaries were mostly in the previtellogenesis stage in all six shrimps at 10 days (GSI = 0.64%, **Figure 4E-1**), at 20 days (GSI = 0.77%, **Figure 4E-2**), one of which also had a few early-stage endogenous vitellogenic oocytes, and in all six shrimps at 30 days (GSI = 0.62%, **Figure 4E-3**).

### Expression Levels of *Liv-SGP-A*, *-B*, and -*C* Following Double-Stranded RNA Injections

**Figure 5:**
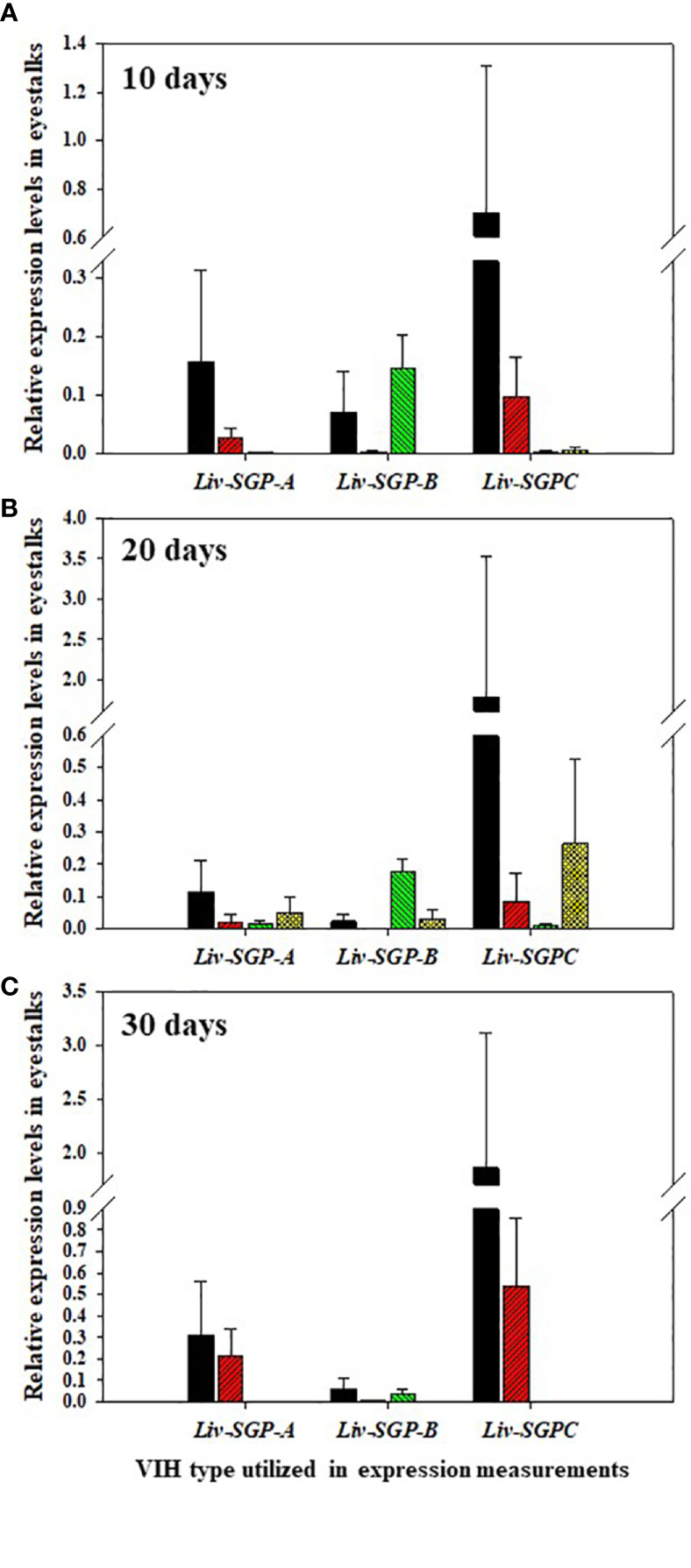
Relative expression levels of *Liv-SGP-A*, *-B*, and *-C* in eyestalks. **(A)** 10 days following a single injection of GFP-dsRNA (n = 5), sgpG-dsRNA (n = 6), sgpC-dsRNA (n = 6), or mixed-dsRNA (n = 6). **(B)** 20 days following a single injection of GFP-dsRNA (n = 6), sgpG-dsRNA (n = 6), sgpC-dsRNA (n = 6), or mixed-dsRNA (n = 5). **(C)** 30 days following a single injection of GFP-dsRNA (n = 4), sgpG-dsRNA (n = 6), sgpC-dsRNA (n = 4), or mixed-dsRNA (n = 4). Results are expressed as the mean ± SEM. Results for each treatment groups are expressed in the bar graphs using the follows colors. Black: GFP-dsRNA group; red: sgpG-dsRNA; green: sgpC-dsRNA; yellow: mixed-dsRNA.

The expression of *Liv-SGP-A*, *-B*, and *-C* was low or not detectable in the eyestalks of subadults (**Figure 5**). In experimental animals 10 days after dsRNA treatment (**Figure 5A**), *Liv-SGP-A* expression were relatively high in the GFP-dsRNA group; however, it was low or not detectable in the other dsRNA treatment groups. Regarding *Liv-SGP-B* expression, levels were relatively higher in the GFP-dsRNA and sgpC-dsRNA groups than in the sgpG-dsRNA and mixed-dsRNA groups. Regarding *Liv-SGP-C* expression, levels were relatively high in the GFP-dsRNA group compared with other dsRNA treatment groups. In experimental animals 20 days after dsRNA treatment (**Figure 5B**), expression levels of *Liv-SGP-A* were relatively high in the GFP-dsRNA group; however, they were low or not detectable in the other dsRNA treatment groups. Regarding *Liv-SGP-B* expression, relatively higher levels were observed in the sgpC-dsRNA group compared to the GFP-dsRNA group (treated as baseline data in this experiment) (**Figure 5B**). Regarding *Liv-SGP-C* expression, levels were relatively high in the GFP-dsRNA group and in the mixed-dsRNA group. In experimental animals 30 days after dsRNA treatment (**Figure 5C**), the expression of *Liv-SGP-A*, and *-C* was relatively high in the GFP-dsRNA group and somewhat high in the sgpG-dsRNA group; however, they were low or not detectable in all other groups. Regarding *Liv-SGP-B* expression, levels were low or not detectable in all dsRNA-treated groups. In this way, *Liv-SGP-A* and *-C* exhibited a similar expression profile, which was to be expected as their corresponding peptides possess a high degree of similarity in terms of amino acid sequence. In general, expression levels of *Liv-SGP-C* were relatively high compared to those of *Liv-SGP-A* and *-B*.

## Discussion

**Figure 6:**
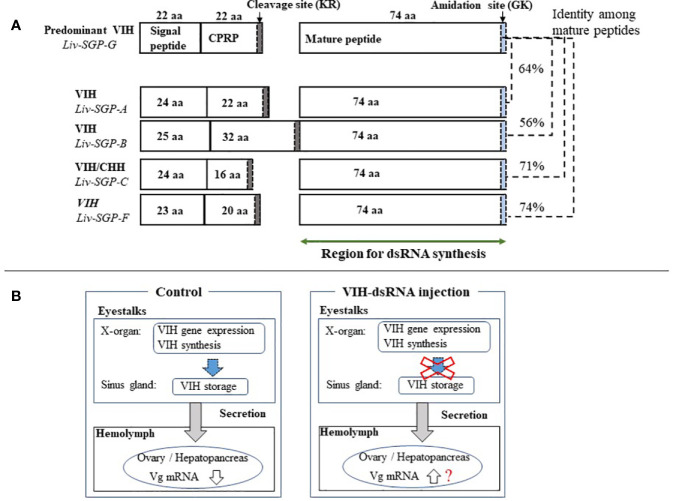
**(A)** Schematic representation of the molecular structure of VIH subtype I peptides in *L. vannamei*. Genetic information for all VIH subtype I peptides (*Liv-SGP-A*, *-B*, *-C*, *-F*, and *-G*) from our previous study was used to construct the drawing (18). The number of amino acid residues (aa) for each corresponding region is shown within each box or above the box in the case of *Liv-SGP-G*. Respective parts are shown as signal peptides, CPRP (CHH precursor-related peptide), and mature peptides. Cleavage sites are indicated by gray shading. Amidation sites are indicated by blue shading. The target region for dsRNA synthesis is indicated by a green arrow. Identity between amino acid sequences corresponding to mature peptide are shown in terms of percentage. **(B)** Schematic representation of VIH regulation on ovarian maturation. VIH that is synthesized and secreted from the eyestalks is thought to fluctuate, thus alternatively suppressing ovarian maturation and allowing it to proceed in nature. Therefore, the suppression of VIH synthesis could be artificially lifted by transcriptional silencing, thus promoting ovarian maturation; however, the knockdown of *vih* transcription alone cannot induce oocyte maturation.

The use of RNAi to silence hormonal transcripts of factors that negatively regulate reproduction offers an opportunity to develop alternatives to eyestalk ablation in shrimp. This is because it is generally known that vitellogenesis-inhibiting hormone (VIH) is produced in the eyestalks and the removal of the source of VIH by eyestalk ablation allows ovarian maturation to occur. In previous work of this laboratory, several sinus gland peptides (SGP) were purified from the eyestalks of subadult *Litopenaeus vannamei*, and six of these were shown to inhibit vitellogenin (VG) gene expression levels using of *Marsupenaeus japonicus* ovary as the *in vitro* incubation system (7). Thereafter, the full-length cDNA of the predominant subtype I VIH in *L. vannamei* was reported, and the ability of recombinant Liv-SGP-G to suppress *vg* expression *in vitro* was demonstrated (17). Thus following, we reported genetic information for all VIH subtype I peptides (Liv-SGP-A, -B, -C, -F, and -G) and the dynamics of their transcription in relation to molting and eyestalk ablation (18). As shown in **Figure 6A**, the molecular structure for VIH subtype I peptides consists of a signal peptide, CHH precursor-related peptide (CPRP), and mature peptide. All VIH subtype I peptides are cleaved at a conserved cleavage site (Lys-Arg) yielding a 74-residue mature peptide having a C-terminal amidation site (Gly-Lys). All mature VIH peptides, which were used as the target region for the preparation of dsRNA synthesis in this study, showed high similarity in terms of deduced amino acid sequence; *Liv-SGP-A*, *-B*, *-C*, and *-F* showed 64, 56, 71, and 74% similarity with *Liv-SGP-G*, respectively (**Figure 6A**). Since these peptides are perceived as isoforms that exhibit the same biological functioning, it is possible that they act in a coordinated fashion, although in terms of their endogenous action, it is unclear whether multiple VIHs act alternately or simultaneously in suppressing ovarian maturation. It is also unclear as to why multiple forms of VIH subtype I exist simultaneously, but if they do have coordinated functioning, it remains necessary to investigate their overall effects on the regulation of ovarian maturation. In this regard, the current study is the first report in shrimp in which transcriptional silencing of a major VIH could be silenced by the administration of dsRNA corresponding to multiple VIH genes.

In this study, a single injection to subadults of sgpG-dsRNA (for *Liv-SGP-G*) silenced the transcription of *Liv-SGP-G* in the eyestalks for at least 30 days. Although we did not include treatment groups for eyestalk ablation and a vehicle control, the observance of positive effects in eyestalk-ablated animals is a well-documented fact (for example, see previously cited studies 19–20); negligible effects can be inferred from the results of the GFP-dsRNA group, the treatment for which was prepared using the same vehicle buffer. As expected, eyestalk ablation induces ovarian maturation and *vg* mRNA expression in subadult females (19, 20), but administration of sgpG-dsRNA did not increase *vg* mRNA expression in this investigation. Injection with multiple dsRNAs corresponding to other VIH subtype I peptides (Liv-SGP-A, −B, −C, −F) also did not promote ovarian maturation. Interestingly, however, the administration of mixed-dsRNAs (for *Liv-SGP-A, −B*, and −*F*) significantly reduced the expression of *Liv-SGP-G* (**Figure 1**). These results show for the first time that administration of dsRNAs can inhibit transcription not only of their specific target genes, but also of homologous genes in shrimp. Perhaps the usage of relatively longer dsRNA in RNAi may achieve the co-transcriptional repression of the specific gene and additionally other homologous genes having high similarity to the basic dsRNA sequences. The possibility of co-suppression by dsRNA was first suggested in the nematode, *Caenorhabditis elegans* (22). Evidence of such a phenomenon was reported in arthropods, specifically in insects [for example, the African malaria mosquito (23)], and as well was observed in shrimp as detailed in this study. Transcriptional silencing generally occurs through long-dsRNA (>200 bp), which is processed into short interfering RNAs (siRNA of 20–30 bp) by endogenous Dicer (an RNase-III-like enzyme) in cells; the resultant siRNA represses transcription of the target gene (24, 25). Therefore, the endogenously produced siRNA may disrupt the expression of homologous genes with short-matched sequences. In *L. vannamei*, the primary structure and encoded sequences have high similarity among the various VIH subtype I peptides; the deduced amino acid sequence of *Liv-SGP-G* has high identity with the other SGP’s as described above. Moreover, CHH-family peptides in general harbor similarity that reflects taxonomic classification; thus, *Penaeus japonicus* and *L. vannamei* show a high degree of similarity, and both species possess several isoforms of VIH subtype I peptide (6, 7). In this way, cross-species effects on VIH activity have been revealed, as Liv-SGP-G peptide inhibited *vg* mRNA expression in the ovary of *P. japonicus* (7). With this in mind, the engineering of a synthetic universal siRNA that targets multiple VIHs in several shrimp species could lead to the development of technology that is valid for implementing artificial maturation in a suite of penaeid shrimp species as a future alternative to eyestalk ablation.

With the above in mind, in this study, we focused on the effects of multiple dsRNA administration on the expression of *Liv-SGP-G*, which is predominant in the experimental species, *L. vannamei*. According to our previous study (18), among the VIH subtype I peptides originating from the eyestalks, corresponding gene expression levels were highest for *Liv-SGP-G* and moderate for *Liv-SGP-C*. On the other hand, the expression of *Liv-SGP-A*, *-B*, and *-F* was low or not detectable throughout molt cycle in adults (of note: *Liv-SGP-E* has been shown to correspond to a peptide identical to Liv-SGP-G, and the peptide translated from *Liv-SGP-D* does not possess VIH activity; hence, there were not examined further) (18). Although expression levels of most of the VIH genes other than *Liv-SGP-G* were low or not detectable in the eyestalks of subadults, their expression profiles yielded useful information on the effects of multiple dsRNA injection as shown in **Figure 5**. These results have revealed that dsRNA corresponding genes other than *Liv-SGP-G*, can also cause the knockdown of their respective genes, and that some of these can also inhibit the transcription of homologous genes (see the **Table 2** for these results represented schematically). In this way, the administration of multiple dsRNA administration is an efficient means of inhibiting a range of VIH expression. These results are expected to be scalable to use in adult animals, and will serve as an important basis for the further development of technology to control reproduction in an artificial environment.

**Table 2:** Schematic representation of the effects of dsRNA administration on the expression levels of *Liv-SGP-A*, *-B*, and *-C* in eyestalks of subadult individuals.

dsRNA treatment type	Levels of Liv-SGP-A			Levels of Liv-SGP-B			Levels of Liv-SGP-C		
	10 days	20 days	30 days	10 days	20 days	30 days	10 days	20 days	30 days
GFP	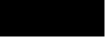	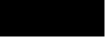	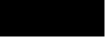	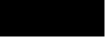	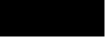	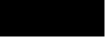	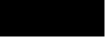	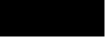	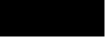
sgpG	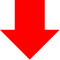	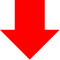	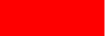	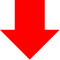	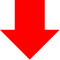	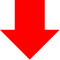	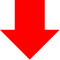	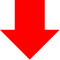	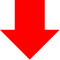
sgpC	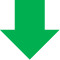	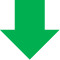	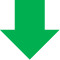	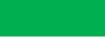	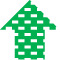	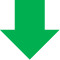	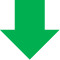	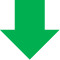	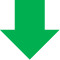
mixed	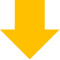	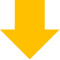	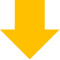	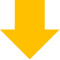	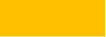	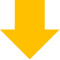	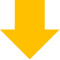	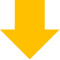	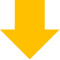

Color for each treatment group corresponds with that utilized in **Figure 5**. Levels of gene expression for each group are shown with the following symbols. GFP-dsRNA group (basal levels): black rectangles; decreased expression: downward-facing solid arrows; increased expression: upward-facing shaded arrow (red, green or yellow rectangle: no change from basal levels).

Previously, the administration of dsRNA with the aim of promoting ovarian maturation, has been used in two of the world’s most important farmed species, *Penaeus monodon* and *L. vannamei* (11, 14–16). In *P. monodon*, a single injection into adult females with GIH-dsRNA at 3 µg g^−1^ body weight knocked down GIH (subtype II) transcription for at least 30 days (11). Nevertheless, it did not promote an increased rate of spawning to the extent that eyestalk ablation did, although it did seem to have some efficacy in wild females as opposed to in domesticated females (14). Results were similar in a study by Feijo et al. (15) using subtype II VIH adult female *L. vannamei*; a single injection with GIH-dsRNA at 2.8 µg g^−1^ body weight knocked down GIH for 37 days, but females did not spawn, and ovarian maturation and increasing *vg* mRNA expression were observed only at 37 days (15). In our previous study, injection with VIH-dsRNA (for *Liv-SGP-G*) at 3 µg g^−1^ body weight suppressed transcription, but did not promote ovarian maturation or increases in endogenous Vg gene expression or concentration even after 20 days (16). Previous work by these and other authors suggests that RNAi could offer an alternative to eyestalk ablation, but with less stimulatory effect than eyestalk ablation. In other words, results obtained thus far have revealed that the knockdown of *vih* transcription alone cannot induce oocyte maturation and spawning in the same manner as eyestalk ablation. Thus, this suggests to us that VIH after being synthesized, is collectively retained in the sinus glands; even after gene expression is suppressed by the administration of dsRNA VIH likely remains in the eyestalks, and can still be secreted into the hemolymph for some time, thereby continuously suppressing ovarian maturation. As we have illustrated in **Figure 6B**, although we believe that endogenous VIH that is synthesized and secreted from the eyestalks fluctuates to thus alternatively suppress ovarian maturation and allow it to proceed in nature, this does not necessarily occur in an artificial environment. Therefore, the suppression of VIH synthesis could be artificially lifted by transcriptional silencing, thus promoting ovarian maturation in a hatchery situation, for example. Perhaps the blocking of actual VIH secretion is also necessary in order to release the suppression of ovarian maturation. Nevertheless, it is still not well-understood how multiple VIHs act to control ovarian maturation, and how one might change their threshold levels artificially. Considering the results obtained thus far, the regulation of ovarian maturation is complicated and is based on the actions of many factors, not only on that of VIH. One of these factors is considered to be the putative vitellogenesis-stimulating hormone (VSH). Although the identity of VSH in shrimp is not clear, various hormonal factors seem to be able to stimulate ovarian maturation (2, 26–29). Hence, the use of RNAi combined with the application of various stimulatory factors may eventually lead to the goal of being able to accelerate ovarian maturation. In *L. vannamei*, injection of synthetic red pigment-concentrating hormone (27), serotonin (2), or serotonin/spiperone (26) can stimulate ovarian maturation, but several injections are required. However, fully matured ovaries that are ready for spawning were not obtained in such studies; this is likely due to the presence of endogenous VIH remaining in the eyestalks. We believe that simultaneous *vih* knockdown and hormonal stimulation may be a means of accelerating ovarian maturation in shrimp. In addition, the engineering of more of potent siRNAs targeting multiple VIHs and the assessment of the effects of combined treatments on ovarian maturation should be investigated in subsequent research.

According to our results, it appeared as though GFP-dsRNA injection positively affected ovarian maturation, because Vg levels in hemolymph were increased at 30 days after the injection of GFP-dsRNA, but not of VIH-related-dsRNA. In actuality, high levels of hemolymph Vg were observed in two individuals after 30 days of the GFP-dsRNA injected group, but there was a large variation in standard error (**Figure 2B**). Perhaps increasing body weight could have caused individual differences in the maturation of subadult females. However, a significant increase in hemolymph Vg levels was shown only at 30 days after GFP-dsRNA injection. We found a similar result in adult female *L. vannamei* in our previous study; injection of adult females with sgpG-dsRNA knocked down *Liv-SGP-G*, but injection with GFP-dsRNA significantly increased GSI only 20 days after (16). Perhaps the surge of VIH in the hemolymph after the knockdown of *vih* transcription delayed ovarian maturation. These results also revealed that subadults have the ability to respond to transcriptional silencing in the same manner as adults. Although the observed levels were low and the effects on ovarian maturation insignificant, *vg* expression levels in the sgpG-dsRNA and sgpC-dsRNA groups were higher than in the GFP-dsRNA group at 20 days after injection. Perhaps this is the result of a *vih* knockdown effect appearing earlier in subadults than in adults. Often, it is the case that ovaries having a large number of immature oocytes will have a similar GSI compared with ovaries having a smaller number of developed oocytes. As in our previous study (19), subadult females that have undergone eyestalk ablation may exhibit developed oocytes even while showing low GSI values. In order to ascertain the effects of each dsRNA treatment on ovarian maturation, we considered it necessary to conduct histological examination of oocyte development as shown in **Figure 4**. Nevertheless, there were no large differences in oocyte development among the various treatments, but there did seem to be more early-stage endogenous vitellogenic oocytes in the mixed-dsRNA group at 10 days as shown in **Figure 4E-1**. On the other hand, RNAi techniques using dsRNA have been used to increase immunity in shrimps; injection of dsRNA for non-specific genes improved the survival of diseased shrimp affected with white spot syndrome virus (25, 30). In our case, injection of GFP-dsRNA may have eventually improved shrimp health conditions in *L. vannamei*. Although we focused only on ovarian maturation, further research focusing not only on ovarian maturation, but also on the immune system, will be valuable for understanding the physiological effects of dsRNA.

In conclusion, we investigated the dynamics of the expression of *Liv-SGP-G* in the eyestalks after injection with dsRNAs corresponding to VIH subtype I peptides (Liv-SGP-A, −B, −C, −F, and −G) and analyzed their effects on ovarian maturation. These results will help improve the current understanding of the biological functioning of VIH subtype I peptides, and in the future may lead to the development of alternatives to eyestalk ablation.

## Data Availability Statement

The datasets presented in this study can be found in online repositories. The names of the repository/repositories and accession number(s) can be found below: https://www.ncbi.nlm.nih.gov/genbank/, LC278950; https://www.ncbi.nlm.nih.gov/genbank/, LC278952; https://www.ncbi.nlm.nih.gov/genbank/, LC278953.

## Author Contributions

BJK and MNW designed the experiments. BJK and ZS performed the experiments and collected raw data. All authors contributed to the article and approved the submitted version.

## Funding

This research was supported by in-house funds provided by the Japan International Research Center for Agricultural Sciences (JIRCAS).

## Conflict of Interest

The authors declare that the research was conducted in the absence of any commercial or financial relationships that could be construed as a potential conflict of interest.
